# Genomic Insights into the Carbon and Energy Metabolism of a Thermophilic Deep-Sea Bacterium *Deferribacter autotrophicus* Revealed New Metabolic Traits in the Phylum *Deferribacteres*

**DOI:** 10.3390/genes10110849

**Published:** 2019-10-26

**Authors:** Alexander Slobodkin, Galina Slobodkina, Maxime Allioux, Karine Alain, Mohamed Jebbar, Valerian Shadrin, Ilya Kublanov, Stepan Toshchakov, Elizaveta Bonch-Osmolovskaya

**Affiliations:** 1 Winogradsky Institute of Microbiology, Research Center of Biotechnology of the Russian Academy of Sciences, 119071 Moscow, Russia; gslobodkina@mail.ru (G.S.); valerianshadrin@gmail.com (V.S.); kublanov.ilya@gmail.com (I.K.); stepan.toshchakov@gmail.com (S.T.); elizaveta.bo@gmail.com (E.B.-O.); 2 Univ Brest, CNRS, Ifremer, LIA1211, Laboratoire de Microbiologie des Environnements Extrêmes LM2E, F-29280 Plouzané, France; Maxime.Allioux@univ-brest.fr (M.A.); Karine.Alain@univ-brest.fr (K.A.); mohamed.jebbar@univ-brest.fr (M.J.)

**Keywords:** autotrophic, thermophile, roTCA cycle, CO oxidation, Fe(III)-reduction, nitrate reduction

## Abstract

Information on the biochemical pathways of carbon and energy metabolism in representatives of the deep lineage bacterial phylum *Deferribacteres* are scarce. Here, we report the results of the sequencing and analysis of the high-quality draft genome of the thermophilic chemolithoautotrophic anaerobe *Deferribacter autotrophicus*. Genomic data suggest that CO_2_ assimilation is carried out by recently proposed reversible tricarboxylic acid cycle (“roTCA cycle”). The predicted genomic ability of *D. autotrophicus* to grow due to the oxidation of carbon monoxide was experimentally proven. CO oxidation was coupled with the reduction of nitrate to ammonium. Utilization of CO most likely involves anaerobic [Ni, Fe]-containing CO dehydrogenase. This is the first evidence of CO oxidation in the phylum *Deferribacteres*. The genome of *D. autotrophicus* encodes a Nap-type complex of nitrate reduction. However, the conversion of produced nitrite to ammonium proceeds via a non-canonical pathway with the participation of hydroxylamine oxidoreductase (Hao) and hydroxylamine reductase. The genome contains 17 genes of putative multiheme c-type cytochromes and “e-pilin” genes, some of which are probably involved in Fe(III) reduction. Genomic analysis indicates that the roTCA cycle of CO_2_ fixation and putative Hao-enabled ammonification may occur in several members of the phylum *Deferribacteres*.

## 1. Introduction

The genus *Deferribacter* belongs to the phylum *Deferribacteres*, which represents a deep lineage in the domain *Bacteria* [1]. Currently, the genus *Deferribacter* comprises four species, *D. thermophilus* [2], *D. desulfuricans* [3], *D. abyssi* [4], and *D. autotrophicus* [5]. Members of the genus *Deferribacter* are thermophilic anaerobes with a Gram-negative type of cell wall, inhabiting deep and shallow marine ecosystems. The metabolism of the *Deferribacter* species is based on the anaerobic respiration of diverse electron acceptors, such as soluble and insoluble forms of Fe(III) [2,4,5], Mn(IV) oxide [2,5], nitrate [2,3,4,5], and elemental sulfur [3,4,5]; fermentative growth is not revealed. The ability to use specific electron acceptors, as well as the possibility for autotrophic growth, varies among species. Their capacity for the dissimilatory reduction of environmentally relevant compounds makes *Deferribacter* a potentially important microbial group in the cycling of iron, nitrogen, and sulfur in deep-sea hydrothermal vents and submarine petroleum reservoirs. Several studies show the abundance of *Deferribacter* among the iron-reducing prokaryotes in various deep-sea hydrothermal systems [6,7,8]. To date, the complete genome has been sequenced only for one representative of *Deferribacter*: *D. desulfuricans* [9]. Genome analysis of this bacterium revealed numerous genes for chemoreceptors, chemotaxis-like systems, and signal transduction machineries that may be linked to versatile energy metabolisms and may provide ecophysiological advantages for *D. desulfuricans* thriving in the physically and chemically fluctuating hydrothermal environment [9]. *D. desulfuricans* is incapable for autotrophic growth and for reduction of Fe(III) and Mn(IV), so genomic determinants of metal reduction in *Deferribacter* remain unexplored. The object of this study, *D. autotrophicus*, isolated from one of the deepest world ocean’s hydrothermal fields [Ashadze field (12°58′21″ N 44°51′47″ W) at a depth of 4100 m, is able to grow chemolithoautotrophically via the oxidation of molecular hydrogen with ferrihydrite, and thus could play a role in the primary production of organic matter in deep-sea ecosystems [5]. Here, we present the results of shotgun de novo sequencing and analysis of the *D. autotrophicus* SL50^T^ genome and provide insight into the carbon and energy metabolism of this bacterium and other species of the phylum *Deferribacteres* with complete sequenced genomes. 

## 2. Materials and Methods 

### 2.1. Genome Sequencing and Assembly

Genomic DNA from a pure culture of *D. autotrophicus* SL50^T^ was extracted with the ISOLATE II™ genomic DNA kit (Bioline, London, United Kingdom), according to manufacturer’s instructions. DNA was quantified by Qubit^®^ dsDNA assay (Thermo Fischer Scientific, Waltham, MA, USA), and quality was assessed using Xpose™ spectrophotometer (Trinean, Gentbrugge, Belgium) and agarose gel electrophoresis. For sequencing of the *D. autotrophicus* genome, both the fragment and long insert (mate-paired) DNA libraries were used. The fragment library was prepared from 500 ng of genomic DNA with the NEBNext Ultra™ DNA library preparation kit (New England Biolabs, Ipswich, MA, USA) according to the manufacturer’s instructions to obtain the mean library size of 400 bp. Mate-paired libraries were prepared with the Nextera™ mate pair library prep kit (Illumina Inc., San Diego, CA, USA) according to the gel-free library preparation protocol, provided by the supplier. Finally, one paired-end and three mate-paired libraries were sequenced with 2 x 250 bp reads with the MiSeq™ personal sequencing system (Illumina Inc.) with standard settings. After sequencing, all reads were subjected to quality filtering and trimming with CLC genomics workbench 9.5 (Qiagen, Hilden, Germany). The 380,899 obtained read pairs were used for assembly. Removal of junction adaptor and identification of true mate-paired reads was performed with the NextClip tool [10], resulting in 1,258,348 true mate-pairs with a mean insert size of 1751 bp. 

Reads were assembled with SPADES 3.9.0 [11]. The obtained contigs were processed manually using the CLC genomics workbench 9.5 package. The resulting assembly consisted of 12 contigs with 2,543,746 bp total length and the N50 value of 319,692 bp. The final assembly coverage was 199.0x.

### 2.2. Genome Annotation

The primary annotation and genomic analyses were performed in an IMG/M v.5.0 analysis system [12] and further refined by NCBI’s (National Center of Biotechnology Information) prokaryotic genome annotation pipeline [13] and manual curation. The study of orthologous genes between *D. autotrophicus* and *D. desulfuricans* megaplasmid was performed by OrthoVenn2 web server [14]. The search and analysis of transposase families was performed by ISSaga web server [15]. Identification and classification of the CRISPR-Cas system was performed by the CRISPRCas Finder web server [16]. The prediction of laterally transferred gene clusters (genomic islands) was performed with the IslandViewer4 web server [17]. Genome visualization was made with the Circos software [18]. For visualization purposes, contigs of *D. autotrophicus* were ordered according to the alignment of the publicly available *D. desulfuricans* genome with the contig ordering tool from the MAUVE genome alignment package [19]. Analysis of the COG (Clusters of Orthologous Genes) functional categories was performed with the eggNOG-Mapper tool on the eggnog Database web server [20]. The analysis of the taxonomic distribution of best blast hits was performed with the AAI (Average Amino-acid Identity) Profiler server [21]. Hydrogenase classification has been checked using the HydDB webtool https://services.birc.au.dk/hyddb/ [22].

### 2.3. Phylogenetic Analysis

The in silico translated proteomes of *D. autotrophicus* and *D. desulfuricans* were searched for molybdopterin oxidorecuctases genes, using BLASTp with known molybdopterin oxidorecuctases sequences from [23] as the queries. Eight sequences were found in each of proteomes. The respective 16 proteins were aligned with the dataset of molybdopterin reductases from [23] (except for a few haloarchaeal sequences) using Mafft v. 7 [24]. The evolutionary history was inferred in MEGA6 [25] by using the maximum likelihood method based on the JTT model [26] and 1000 bootstrap replications. All positions with less than 95% site coverage (gaps, missing data, and ambiguous bases) were eliminated. There was a total of 460 amino acid positions in 129 sequences in the final dataset. 

### 2.4. Growth Experiments on Carbon Monoxide Utilization

*Deferribacter autotrophicus* SL50^T^ was cultivated in 17 mL Hungate tubes (Bellco Glass Inc., Vineland, NJ, USA) filled with 5 mL of the liquid medium. The medium’s composition and preparation are described in [5], the organic electron donors (lactate, acetate) were omitted. The medium contained 100 mg/L of yeast extract (Difco^TM^, Seneca, KS, USA) and 10 mM KNO_3_ (Sigma-Aldrich, p.p.a., St. Louis, MI, USA) as an electron acceptor. The gas phase was filled with CO_2_ (100%, 1.0 atm, analytical grade, JSC MGPZ, Moscow, Russia). Carbon monoxide (gas, analytical grade, JSC MGPZ) was added to each test tube by syringe to obtain the final CO concentration of 10% (*v*/*v*). Cultivation was performed at 60 °C. The growth of bacteria was determined by direct counting of the cells with a phase-contrast microscope (Olympus CX41, Japan) and a counting chamber. CO, H_2_, alcohols, and volatile fatty acids were analyzed by gas chromatography, as described in [27]. Ammonium was determined by the phenol-hypochlorite reaction; nitrate was analyzed by HPLC, as described in [28]. In growth experiments, series of the Hungate tubes were incubated in parallel, and for each measuring point, three independent test tubes were used. All experiments were repeated three times. Mean square deviations for the six measurements are presented throughout the paper.

### 2.5. Data Availability

The draft genome of *D. autotrophicus* SL50^T^ was deposited in DBJ/EMBL/GenBank under the accession nos. VFJB00000000, PRJNA546543, and SAMN11960996 for the Genome, Bioproject, and Biosample, respectively.

## 3. Results and Discussion

### 3.1. General Genome Properties, Genome Mobility, and Genomic Islands

**Figure 1:**
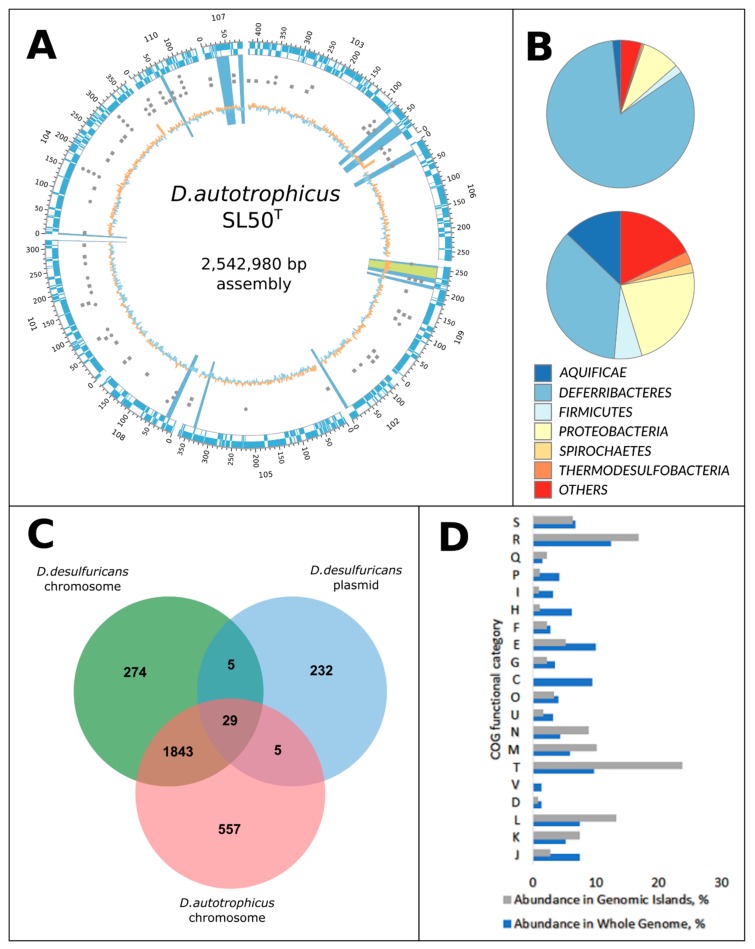
Genomic features of *Deferribacter autotrophicus* SL50^T^. (**A**) Circular map of the genomic features of *D. autotrophicus*. Rings from outside to inside: contig ID (corresponding to the locus number suffix, e.g., Ga0300908_101); genomic coordinates in the corresponding contig; plus-strand CDS (light blue); minus-strand CDS (light blue); GI-related features—tRNAs (grey circles) and transposases (grey squares); GC-content, relative to the average GC-value. Genomic islands are shown as light-blue sectors; prophage is shown as the light green sector. Contigs of *D. autotrophicus* were ordered according to the alignment of the publicly available *D. desulfuricans* genome (NC_013939) with the contig ordering tool from the MAUVE genome alignment package [19]; (**B**) Genome-wide (top) and genomic islands (bottom) taxonomic distribution of proteins best blast hits; (**C**) Venn diagram of protein orthologs shared by the assembly of *D. autotrophicus* chromosome and plasmid of *D. desulfuricans*. Orthology analysis of was performed with OrthoVenn2 web server [14] using 0.01 blast e-value and 1.5 orthoMCL grain value. Numbers indicate shared or unique protein clusters and singleton proteins. (**D**) Distribution of COG functional categories in the genome (blue bars) and in genomic islands (grey bars). Functional categories are represented as follows: Amino acid transport and metabolism [E], Carbohydrate transport and metabolism [G], Cell cycle control, cell division, chromosome partitioning [D], Cell Motility [N], Cell wall/membrane/envelope biogenesis [M], Coenzyme transport and metabolism [H], Defense mechanisms [V], Energy production and conversion [C], Function unknown [S], General function prediction only [R], Inorganic ion transport and metabolism [P], Intracellular trafficking, secretion, and vesicular transport [U], Lipid transport and metabolism [I], Mobilome: prophages, transposons [X], Nucleotide transport and metabolism [F], Posttranslational modification, protein turnover, chaperones [O], Replication, recombination, and repair [L], Secondary metabolites biosynthesis, transport, and catabolism [Q], Signal transduction mechanisms [T], Transcription [K], Translation, ribosomal structure, and biogenesis [J].

Draft genome of *D. autotrophicus* SL50^T^ consists of 2,542,980 nucleotides and has a GC content of 32.62%. Annotation with PGAP [13] resulted in prediction of 2504 genes, 2451 of which are protein-coding genes, 53 RNA genes, and 23 pseudogenes. The genome of *D. desulfuricans* harbors a 300 Mbp megaplasmid. With respect to that fact, we performed additional analysis of our data to identify plasmid-related contigs in our assembly. Nevertheless, neither analysis of the assembly coverage, nor the alternative assembly with plasmidSPAdes [29] revealed the presence of any extrachromosomal elements in the *D. autotrophicus* SL50^T^ genome. On the other hand, the genome of *D. autotrophicus* SL50^T^ is almost 300 Mbp larger than the chromosome of *D. desulfuricans* (Table 1). However, this could not be explained by the integration of *D. desulfuricans* megaplasmid into the chromosome due to the fact that only five orthologues of genes encoded by the megaplasmid were detected in the *D. autotrophicus* genome by the OrthoVenn2 web server and ProteinOrtho orthology detection tool (Figure 1C, Supplementary Table S1) [14,30]. In turn, analysis of the transposase families present in the *D. autotrophicus* genome revealed a high level of diversity of IS elements, indicating that a significant number of HGT events occurred during the evolution of this species. Analysis of orthologous genes clusters by the OrthoVenn2 web server [14] showed that *D.autotrophicus* and *D.desulfuricans* genomes share 1877 gene clusters and possess 557 and 511 species-specific CDS, respectively (Figure 1C).

Genetic immunity in *D. desulfuricans* SSM1 is ensured by the I-B type of the CRISPR-Cas system [4,31]. In turn, *D. autotrophicus* possesses only two genes of I-B type Cas clusters (Cas6—FHQ18_10400 and Cas8b1/Cst1—FHQ18_10405), neighboring two transposase genes, which possibly disrupted the other part of the I-B type effector complex. Taken together, these observations suggest that the increased chromosome size of *D. autotrophicus* is a result of the loss of genomic immunity systems and subsequent numerous lateral gene acquisition events during the evolution of *D. autotrophicus*. In accordance with this assumption, the prediction of laterally transferred genes with the IslandViewer4 web server [17] showed that the *D. autotrophicus* SL50^T^ genome possesses 12 genomic islands of 164 kb total length, which is twice the number and total length of the GIs in the genome of *D. desulfuricans* (Table 1 and Table S2, Figure 1D). Analysis of the COG functional categories of genes, located in the predicted GIs, showed that apart from the “L—Replication, recombination, and repair” category, largely represented by integrases, transposases, and other genome mobility-related genes, almost one-third of genomic islands were represented by genes involved in the regulation of transcription and signal transduction (Figure 1B, Table S3), ensuring attenuation of gene regulation circuits to changing environmental conditions. Further, 19% of the predicted laterally transferred genes were attributed to cell membrane biogenesis and cell motility functions (Figure 1D, Table S3). Therefore, it might be speculated that genomic islands, acquired by *D. autotrophicus*, play an important role in the adaptation of *D. autotrophicus* cells for living in both the sharp and flexible physicochemical gradients of deep-sea hydrothermal vents. In that aspect, *D. autotrophicus* genome is expectedly similar to *D. desulphuricans*, also significantly enriched by signal transduction, motility, and chemotaxis-related genes [9]. Nevertheless, analysis of PFAM domains, involved in environmental sensing, showed that despite the genome of *D. autotrophicus* being slightly smaller than the genome of *D. desulfuricans*, it possesses a higher number of genes, related to signal transduction systems (Table S4). Observation that at least 13% of these genes are located in detected genomic islands supports the hypothesis regarding the important role of laterally acquired genes in regulatory networks of *D. autotrophicus*.

Genome-wide analysis of the taxonomic distribution of the best blast hits (BBHs) of proteins showed a similar overall BBH pattern to *D. desulfuricans*, which is significantly enriched by genes with the closest orthologues in *Deltaproteobacteria*, *Firmicutes*, and *Aquificae*, showing 54.7, 48.6, and 62.3% mean AAI, respectively [9]. Remarkably, the BBH taxonomy affiliation of more than 40% of proteins encoded in the genomic islands points to an extensive gene flow between these taxa. Interestingly, genomic islands were enriched by the genes, encoding proteins with the best orthologs from phylum *Spirochaetes* (Figure 1A, Table S5). Although this phylum has only a few cultivated thermophilic members [32,33,34], its representatives might have a significant input to the hydrothermal field’s gene pool.

### 3.2. Central Metabolism

*Deferribacter autotrophicus* can anaerobically oxidize formate, acetate, lactate, pyruvate, malate, fumarate, succinate, maleinate, maltose, and molecular hydrogen with nitrate, elemental sulfur, Fe(III), or Mn(IV) as electron acceptors [5]. Organic acids could be utilized as energy and carbon sources via the oxidative tricarboxylic acid cycle (TCA). The genome of *D. autotrophicus* contains all the genes encoding TCA enzymes, including: citrate synthase (FHQ18_06115), aconitate hydratase (FHQ18_06070), isocitrate dehydrogenase (FHQ18_05945), 2-oxoglutarate/2-oxoacid ferredoxin oxidoreductase (FHQ18_01050, FHQ18_01055; FHQ18_05965, FHQ18_05970, FHQ18_05975, FHQ18_05980; FHQ18_06355, FHQ18_06360, FHQ18_06365, FHQ18_06370), succinyl-CoA ligase (FHQ18_05955, FHQ18_05960), succinate dehydrogenase (FHQ18_05990, FHQ18_05995), fumarate hydratase (FHQ18_05155), and malate dehydrogenase (FHQ18_05950). Acetate can enter TCA after activation to acetyl-CoA either in a single-step reaction, catalyzed by an acetyl-CoA synthetase (FHQ18_05360; FHQ18_07465), or (at high acetate concentration [35]) in a two-step reaction, catalyzed by acetate kinase (FHQ18_04100) and phosphate acetyltransferase (FHQ18_04095). The genome of *D. autotrophicus* contains the full set of genes of the Embden–Meyerhof–Parnas (EMP) pathway and the non-oxidative branch of the pentose phosphate pathway (PP). The EMP and PP pathways in *D. autotrophicus* probably also operate in the reverse direction and are involved in the synthesis of cell material during autotrophic growth. 

*Deferribacter autotrophicus* is capable of using molecular hydrogen as an energy source. The genome of *D. autotrophicus* contains two gene clusters encoding membrane-bound [NiFe]-hydrogenases, belonging to the Group 1f and the Group 1c [NiFe]-hydrogenases, respectively, which are likely to be involved in the H_2_-uptake. The first gene cluster (FHQ18_02875, FHQ18_02880, FHQ18_02885, and FHQ18_02890) encodes three hydrogenase subunits (HyoS, HyoL, and HyoE) and a hydrogenase maturation protease. All three subunits are strongly conserved (84% to 93% amino acid identity) with those of the [NiFe]-hydrogenase of *D. desulfuricans*, the bacterium capable of H_2_ utilization. The [NiFe] Group 1f-hydrogenase class is polyphyletic and has been poorly studied. It may contain several loosely-related hydrogenases with different roles, such as oxidative stress defense in *Geobacter sulfurreducens* [36] or consumption of sub-atmospheric concentrations of H_2_ in *Acidobacterium ailaaui* [37]. We can hypothesize that this hydrogenase could be involved in hydrogenotrophic respiration of nitrate or sulfur. The second cluster (FHQ18_08580, FHQ18_08560, 65, 70, and 75) contains the genes of a hydrogenase maturation protease and four subunits (HybO, HybA, HybB, and HybC) of the [NiFe]-hydrogenase, which couples hydrogen oxidation in the periplasm to reduction of the inner membrane quinone pool. Notably, genes of the Fe-S-cluster-containing protein HybA and integral membrane subunit HybB are absent in *D. desulfuricans*, indicating the incompleteness of the given hydrogenase complex in this microorganism. HybABCO is structurally similar to the hydrogenase 2 of *Escherichia coli*, which is expressed under anaerobic conditions and is sensitive to oxygen and nitrate [38,39]. The Group 1c [NiFe]-hydrogenases class is described to be involved in anaerobic hydrogen uptake with terminal electron acceptors such as metals, fumarate, and sulfate. In *D. autotrophicus*, HybABCO might be used for Fe(III) reduction. The [NiFe]-hydrogenase maturation system HypABCDEF in *D. autotrophicus* is encoded in FHQ18_04530, FHQ18_04535, FHQ18_04540, FHQ18_05795, FHQ18_05800, and FHQ18_06255.

### 3.3. Autotrophic CO_2_ Fixation

**Figure 2:**
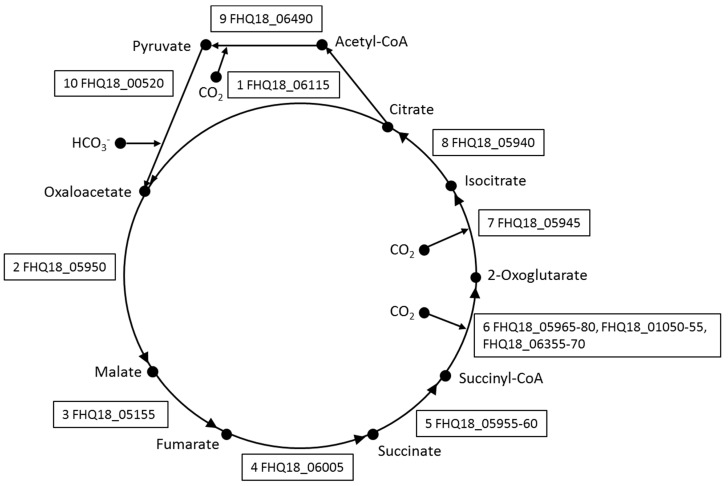
A reversed oxidative TCA cycle in *D. autotrophicus* reconstructed from the genomic analysis. Enzymes: 1, citrate synthase; 2, malate dehydrogenase; 3, fumarate hydratase; 4, fumarate reductase / succinate dehydrogenase; 5, succinyl-CoA synthetase; 6, 2-oxoglutarate ferredoxin oxidoreductase; 7, isocitrate dehydrogenase; 8, aconitase; 9, pyruvate synthase; 10, pyruvate carboxylase.

*Deferribacter autotrophicus* grows autotrophically using CO_2_ as the carbon source, molecular hydrogen as an electron donor, and ferric iron as an electron acceptor [5]. In the *D. autotrophicus* genome, we did not find genes encoding the key enzymes of six microbial carbon fixation pathways, viz. ribulose 1,5-bisphosphate carboxylase (Calvin-Benson cycle), carbon monoxide dehydrogenase/acetyl-CoA synthase complex (reductive acetyl-CoA pathway), ATP-citrate lyase and citryl-CoA lyase (two variants of the reductive tricarboxylic acid cycle), 4-hydroxybutyryl-CoA dehydratase (3-hydroxypropionate/4-hydroxybutyrate and dicarboxylate/4-hydroxybutyrate cycles), and malonyl-CoA reductase (3-hydroxypropionate bi-cycle). However, the genome of *D. autotrophicus* contains a gene cluster that encodes five enzymes of the reductive TCA cycle (FHQ18_05940-06005). The fumarase gene FHQ18_05155 was located distantly from the cluster. However, the in-silico translation of a pseudogene FHQ18_05935, located within this cluster, was predicted as a fumarase. Besides the four subunit 2-oxoglutarate/2-oxoacid ferredoxin oxidoreductase (FHQ18_05965-80) encoded by genes within the cluster FHQ18_05940-06005, two additional copies of the enzyme are encoded by distantly located genes: two subunits enzyme FHQ18_01050- 55 and four subunits enzyme FHQ18_06355-70. The closest characterized homologs of the first of these genes are from archaea *Sulfolobus* [40] and *Aeropyrum* [41] (50.2 and 47.8%/32.3 and 27.6% AA sequence identity for beta and alpha subunits, respectively, with almost 100% coverage) and act in the opposite direction, decarboxylating pyruvate and 2-oxoglutarate, respectively, thereby acting as a part of the oxidative TCA. This is a possible point of the regulation of the direction of the cycle. Moreover, the genome of *D. autotrophicus* contains one gene of citrate synthase (CS) (FHQ18_06115). Under chemolithoautotrophic conditions, citrate synthase can cleave citrate adenosine triphosphate independently into acetyl coenzyme A and oxaloacetate [42,43]. Therefore, the TCA cycle operated in the reductive direction with the reverse reaction of CS [42,43] (Figure 2). Acetyl-CoA is reductively carboxylated to pyruvate by ferredoxin-dependent pyruvate synthase (FHQ18_06490). The presence of genes of anaplerotic enzymes, pyruvate carboxylase (FHQ18_00520), phosphoenolpyruvate carboxykinase (FHQ18_07095), and oxaloacetate- decarboxylating malate dehydrogenase (malic enzyme, FHQ18_00980, FHQ18_05150), indicates the possibility to maintain the level of the cycle intermediates. Considering all these observations, *D. autotrophicus* CO_2_ fixation can occur, most likely, via the recently discovered reversible TCA cycle (“reversed oxidative TCA cycle”) [42,43] (Figure 2). 

Among representatives of the phylum *Deferribacteres* with sequenced genomes, the capacity for autotrophic growth was experimentally shown for *D. autotrophicus* and suggested for *Geovibrio thiophilus*, which can be repeatedly transferred with H_2_ and S^0^ in the absence of organic compounds [44]. Like *D. autotrophicus*, the genome of *G. thiophilus* did not contain genes encoding the key enzymes of the six microbial carbon fixation pathways (see above) but did contain the complete set of genes of TCA cycle. The citrate synthase (CS) of *G. thiophilus* (WP_128467229) has 77% amino-acid sequence identity with the CS of *D. autotrophicus*. These findings suggest a similar mode of CO_2_ assimilation in *D. autotrophicus* and *G. thiophilus* and make both microorganisms promising candidates for the experimental testing of the recently proposed reversible TCA cycle. The prediction of roTCA cycle based entirely on bioinformatic data is not currently possible. For instance, the genome of *Deferribacter desulfuricans* contains all genes of TCA cycle, including putative CS, which is highly similar (98% protein identity) to CS of *D. autotrophicus*, but *D. desulfuricans* is incapable for autotrophic growth tested with several electron acceptors [3,9]. It was suggested that the absence of ATP-dependent citrate lyase is the reason of inability of *D. desulfuricans* to grow autotrophically [9], but *D. autotrophicus* also lacks genes encoding this enzyme, thus failure of *D. desulfuricans* for autotrophic growth could be caused by other factors. 

### 3.4. Carbon Monoxide Utilization

**Figure 3:**
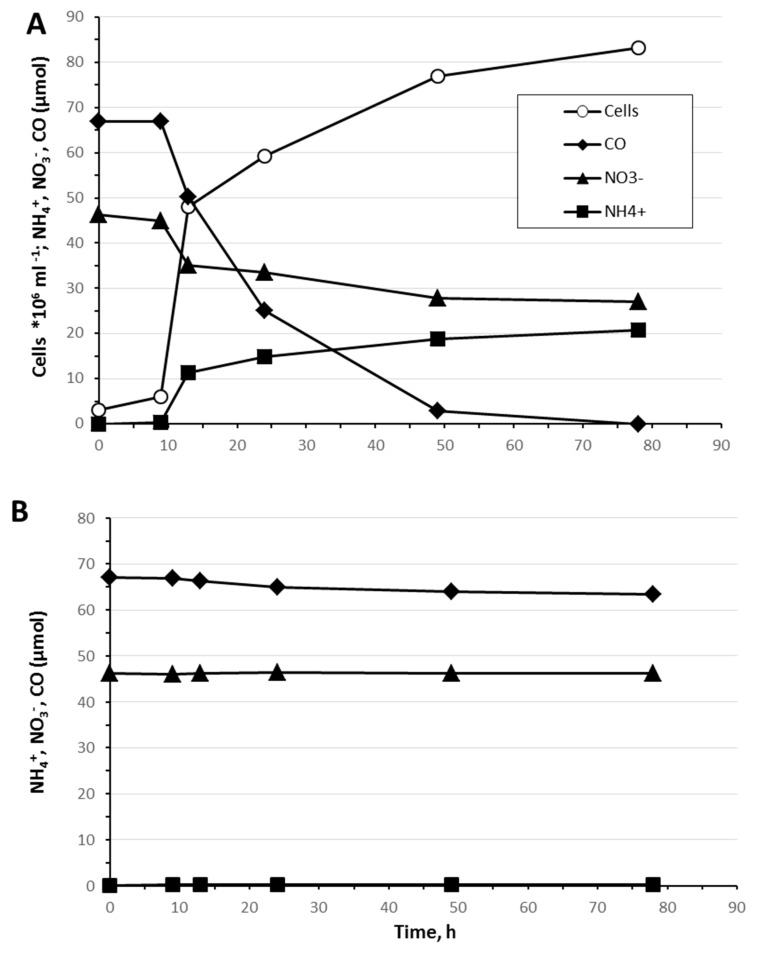
(**A**) Growth of *D. autotrophicus* with CO as an electron donor and nitrate as electron acceptor. (**B**) In non-inoculated controls, the concentration of CO and nitrate was not changed, and no ammonium accumulation was detected.

The ability of *D. autotrophicus* to utilize carbon monoxide (CO) has not been tested in the original description [5]. We found that the genome of *D. autotrophicus* contains two genes that encode anaerobic [Ni, Fe]-containing carbon-monoxide dehydrogenase (CODH), which prompted us to experimentally verify CO utilization. Indeed, *D. autotrophicus* grew in batch cultures in a liquid anaerobic cultivation medium supplemented with CO (10% *v*/*v* in the gas phase) as an electron donor and nitrate (10 mM) as an electron acceptor (Figure 3A). Under these conditions, 100 mg/L of yeast extract was required for sustainable growth. No growth on CO occurred with Fe(III) citrate, ferrihydrite, Mn(IV), elemental sulfur, or in the absence of an electron acceptor. In nitrate-grown cultures, maximal cell density was 8.3 × 10^7^ cells/mL, and the specific growth rate was 0.225 ± 0.004 h^−1^ (doubling time: 3.15 h). Cell growth was coupled to the removal of CO and nitrate and the accumulation of ammonium. The nitrate was reduced to ammonium: from 3.79 ± 0.53 mM of NO_3_^−^ consumed, 3.69 ± 0.51 mM of NH_4_^+^ was produced. No nitrite, N_2_, or N_2_O were detected in the logarithmic or stationary phases of growth. In the non-inoculated chemical controls, the initial CO and nitrate concentration was not changed, and no ammonium accumulation was detected during incubation at 600 °C for 78 h (Figure 3B). Carbon monoxide was presumably oxidized to CO_2_ (not quantified). Other possible products of CO oxidation, including acetate, ethanol, and C_3_–C_5_ volatile fatty acids and alcohols, were not found in the cultivation medium. No production of molecular hydrogen was observed in any phase of growth. The maximal tested CO concentration that supported growth was 50% *v*/*v* in the gas phase. 

The ratio of CO consumed to ammonium produced was 3.77 ± 0.56. This CO/NH_3_ ratio is close to the theoretical ratio for the following reaction:

4CO + NO_3_^−^ + 2H_2_O = 4CO_2_ + NH_3_ + OH^−^
[CO/NH_3_] = 4.00; Δ*G*°’ = −593.6 kJ per reaction; Δ*G*°’ = −148.4 kJ·mol^−1^ CO.

The oxidation of carbon monoxide with nitrate as an electron acceptor has been reported for *Moorella thermoautotrophica* and *Moorella thermoacetica* [45]. In these homoacetogenic bacteria, NO_3_^−^ inhibits CO utilization under autotrophic conditions, but growth on CO, coupled to nitrate reduction to nitrite and ammonium, can be obtained in the medium supplemented with high concentrations of organic compounds such as vanillate (5 mM), syringate (5 mM), or yeast extract (1.0 g/L), which are used as carbon sources [45]. In contrast, *D. autotrophicus* cannot utilize CO in the absence of nitrate and does not require large amounts of organics for CO-dependent growth. 

**Figure 4:**
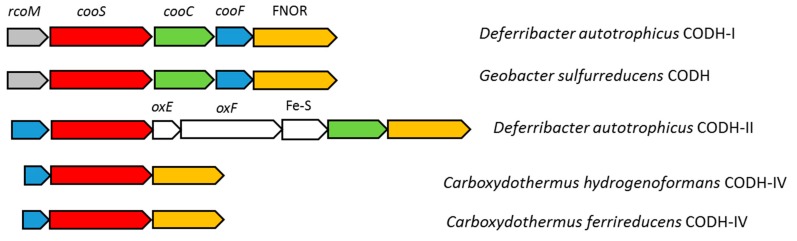
Carbon monoxide dehydrogenase gene clusters in *D. autotrophicus* and similar gene clusters present in other organisms. The genes of the following proteins are highlighted: rcoM, CO-sensing transcriptional regulator; coos, CO dehydrogenase catalytic subunit; cooC, accessory protein; cooF, Fe–S cluster—binding protein; FNOR, FAD–NAD oxidoreductase; oxE and oxF, NADH:ubiquinone oxidoreductase subunits E and F; Fe-S, 4Fe-4S dicluster protein.

**Figure 5:**
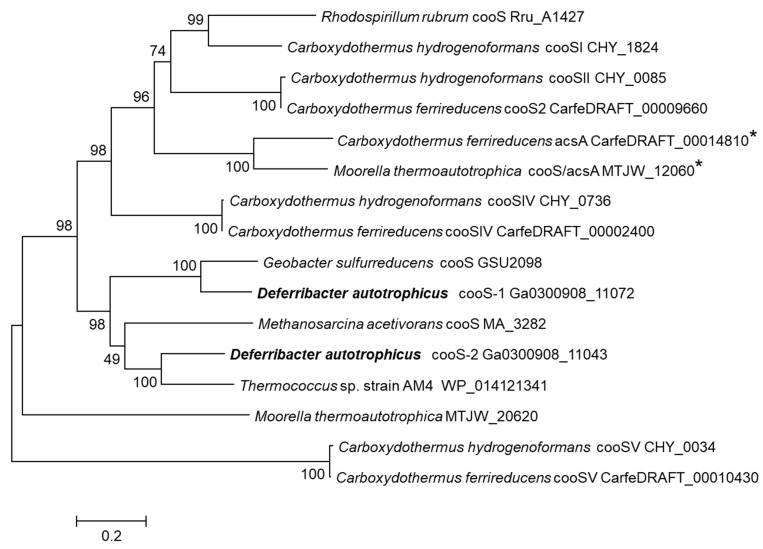
The phylogenetic tree of *D. autotrophicus* [Ni,Fe]-CODHs. The tree was constructed by the maximum likelihood method using MEGA7 [51] at default parameters after aligning the sequences with the built-in ClustalW at default parameters. Enzymes involved in the Wood–Ljungdahl pathway of acetyl-CoA synthesis from C1 units are marked with an asterisk.

In the *D. autotrophicus* genome, [Ni, Fe]-CODH-coding genes are localized in two differently organized gene clusters (Figure 4). Cluster CODH-I consists of the CO-sensing transcriptional regulator RcoM (FHQ18_12160); catalytic CODH subunit CooS (FHQ18_12155); an accessory protein CooC, involved in ATP-dependent Ni-insertion into CODH (FHQ18_12150); iron–sulfur-binding protein CooF, putatively transferring electrons from CooS via the Fe–S clusters (FHQ18_12145); and FAD–NAD(P) oxidoreductase FNOR, probably involved in electron transfer to the quinone pool (FHQ18_12140). A similar organized gene cluster has been described for *Geobacter sulfurreducens*, which utilizes CO with fumarate as an electron acceptor and has single monofunctional CODH [46]. Amino-acid identity between the *D. autotrophicus* CooS FHQ18_12155 and CooS of *G. sulfurreducens* is 74%, and they are grouped together on the phylogenetic tree (Figure 5). The *D. autotrophicus* cluster CODH-II includes genes of CooF (FHQ18_12005), CooS (FHQ18_12010), CooC (FHQ18_12030), and FNOR (FHQ18_12035), as well as three genes encoding proteins annotated as NADH:ubiquinone oxidoreductase subunits E and F (FHQ18_12015-20) and the 4Fe-4S dicluster protein (FHQ18_12025). Genes of the putative oxidoreductases from COG1905 and COG1894 are often present in the genomic context of [Ni, Fe]-CODH-containing loci. However, functions of the respective proteins in CO metabolism are not clear [47]. Notably, genes encoding the putative NADH:quinone-oxidoreductase subunits E and F, along with the 4Fe-4S domain protein (CarfeDRAFT _00002360 _00002350 _00002340, IMG annotation), are part of the CODH-IV-containing genomic locus in *Carboxydothermus ferrireducens* that is capable of CO oxidation with fumarate, 9,10-anthraquinone-2,6-disulfonate, or Fe(III) as an electron acceptor [27,48]. Among the [Ni, Fe]-CODHs of carboxydotrophic microorganisms, CooS-II of *D. autotrophicus* has the highest amino-acid identity (66%), with the CooS of *Thermococcus* sp. strain AM4 belonging to clade E of anaerobic carbon-monoxide dehydrogenases [47,49,50]. The gene encoding homolog of CO- and redox-sensing regulator protein CooA (FHQ18_06185) is present in the *D. autotrophicus* genome but is located separately from the two CODH clusters. Genes encoding *cox*-type aerobic Mo- and Cu-containing CODHs belonging to the xanthine oxidase family were not identified in the *D. autotrophicus* genome. 

The functional role of two *D. autotrophicus* CODHs is difficult to predict from the genomic data. Considering the high amino-acid identity of the CooSI of *D. autotrophicus*, a single monofunctional CooS of *G. sulfurreducens* and the same operon formula of their gene clusters, it is highly likely that in *D. autotrophicus*, CODH-I is involved in CO oxidation. The biological role of CODH-II of *D. autotrophicus* is unclear. Its genomic context resembles the CODH-IV gene clusters of *Carboxydothermus hydrogenoformans* and *Carboxydothermus ferrireducens*, which are thought to be involved in defense against oxidative stress [52]. However, the *D. autotrophicus* CODH-II cluster lacks the gene encoding rubrerythrin, which performs the final step of the detoxification–hydrogen peroxide reduction. Protein sequence identities between NADH:ubiquinone oxidoreductase subunits of *D. autotrophicus* and subunits HndA, HndB, and HndC of electron-bifurcating hydrogenase of *Desulfovibrio fructosovorans* [53] were 35, 28, and 57%, respectively, thus leaving open the question of similar catalytic mechanisms of these enzymes. 

A search for *cooS* genes among other representatives of the phylum *Deferribacteres* with sequenced genomes did not yield positive results. Thus, to date, *D. autotrophicus* is the only known representative of the phylum *Deferribacteres* capable of CO metabolism.

### 3.5. Nitrate Reduction

*Deferribacter autotrophicus* reduces nitrate to ammonium in the course of growth with organic electron donors [5]. Microbial dissimilatory nitrate reduction to ammonium proceeds via the formation of NO_2_^−^ by rather well-characterized Nap- or Nar-type enzyme complexes, but the step of nitrite reduction to ammonium differs in many microorganisms and is not completely understood [54]. 

**Figure 6:**
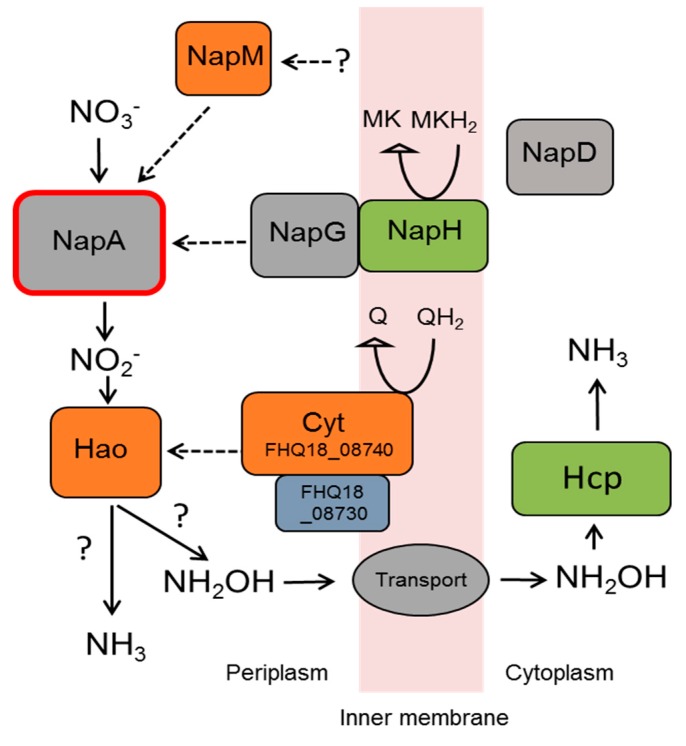
The putative enzymatic system of nitrate reduction to ammonium in *D. autotrophicus*. See explanations in the text.

The genome of *D. autotrophicus* contains a 9-gene cluster (FHQ18_08700- FHQ18_08740) relevant to dissimilatory nitrogen metabolism. This cluster comprises a five-gene locus of the Nap-type nitrate reduction complex and four genes probably involved in the reduction of nitrite. The Nap-type system encoded by *napMADGH* (FHQ18_08700–FHQ18_08720) includes the small periplasmic cytochrome NapM, the catalytic molybdopterin-binding subunit NapA, the chaperone NapD, and ferredoxin-containing electron transfer subunits, NapG and NapH. The Nap system of *D. autotrophicus* lacks the membrane-bound quinol-oxidizing tetraheme cytochrome NapC and diheme cytochrome NapB, which is also a characteristic of the Nap systems of deep-sea nitrate-ammonifying thermophiles *Dissulfuribacter thermophilus* (class *Deltaproteobacteria*), *Thermosulfurimonas dismutans* (class *Thermodesulfobacteria*) [28,55], and *Caldithrix abyssi*, (phylum *Calditrichaeota*) [56]. The reduction of the produced nitrite to ammonium does not proceed via the canonical Nrf system because the gene, encoding the key enzyme of this pathway (i.e., pentaheme cytochrome *c* nitrite reductase NrfA), was not found in the genome. It has been proposed that the Nrf complex could be replaced by an ammonification pathway based on the reversibly acting hydroxylamine oxidoreductases (Hao) [57]. In *D. autotrophicus*, the enzymes that are presumably a part of this pathway are encoded by other four genes of the 9-genes cluster (FHQ18_08725-40). These proteins include periplasmic octaheme hydroxylamine oxidoreductase (Hao), a small membrane-bound protein of unknown function, hydroxylamine reductase (Hcp), and decaheme cytochrome *c* with one transmembrane helix. We hypothesize that the reduction of nitrite to ammonium proceeds by the action of cytochrome-containing hydroxylamine oxidoreductase (Hao) and hydroxylamine reductase (Hcp) via the formation of hydroxylamine as an intermediate (Figure 6). It is also possible that Hao directly reduces NO_2_^−^ to NH_3_ without the help of Hcp. Alternatively, octaheme tetrathionate reductase (Otr) could be involved in nitrite reduction [58]. The genome of *D. autotrophicus* contains the *otr* gene (FHQ18_05250) encoding protein, which seems to be localized in the periplasmic space, as indicated by the presence of a signal peptide. Ammonium transporters are encoded by the genes FHQ18_06975 and FHQ18_08825, the latter located close to the proposed *nap-hao* gene cluster. The genome of *D. autotrophicus* does not contain the *qcrABC* gene cluster that encodes the menaquinol-cytochrome *c* reductase complex involved in nitrate respiration in some *Epsilonproteobacteria* [59,60]. Interestingly, the genome of *D. desulfuricans*, which reduces nitrate to nitrite but does not produce ammonium, also contains a gene cluster (DEFDS_1817-1823) encoding NapMADGH, Hao, and a small membrane-bound protein of an unknown function similar to FHQ18_08730 of *D. autotrophicus*. However, *D. desulfuricans* does not contain homologs of the transmembrane decaheme cytochrome *c* and *otr* genes present in *D. autotrophicus*, and the Hcp-coding gene is distantly located from the *nap* gene cluster. The membrane-bound nitrate-reducing enzymes (Nar: DEFDS_2086-89) present in the genome of *D. desulfuricans* are absent in *D. autotrophicus*. This absence is important, since the Nar enzyme complex can generate the proton motive force (pmf), unlike Nap and Hao enzymes, making the *D. autotrophicus* nitrate/nitrite reduction dependent on other pmf-driving mechanisms. 

The genomes of all genome-sequenced cultivated members of the phylum *Deferribacteres* contain the *napMADGH* gene cluster (Table 1), but the reduction of nitrite to ammonium in *Denitrovibrio acetiphilus* and *Geovibrio thiophilus* apparently occurs without the participation of NrfA, as in the *Deferribacter* species.

### 3.6. Iron Reduction

*Deferribacter autotrophicus* reduces the soluble and insoluble forms of Fe(III) during the course of growth with organic electron donors or molecular hydrogen [5]. The biochemical mechanisms and pathways of dissimilatory iron reduction are diverse, and currently no universal genomic determinants of this process are known. However, it is clear that in many organisms, *c*-type cytochromes play a key role in electron transfer out of the cell to the extracellular electron acceptor [61,62]. Another suggested mechanism for extracellular electron transport involves electrically conductive pili (‘e-pili’). However, many Fe(III) reducers lack e-pilin genes [63]. 

In the genome of *D. autotrophicus*, we did not find homologs of the outer- or inner-membrane cytochromes, MtrA, MtrC, OmcS, CymA, ImcH, and CbcL, and the porin-like outer-membrane proteins, MtrB and OmbB, which have been shown to determine electron transfer in the model Fe(III)-reducing bacteria, *Shewanella oneidensis*, and *Geobacter sulfurreducens* [64,65,66,67,68]. The *D. autotrophicus* genome contains 17 genes that encode putative multiheme *c*-type cytochromes with a number of heme *c*-binding motifs from two to 28 (Table S6). Among these, 10 genes encoded transmembrane proteins, and five genes encoded periplasmic proteins possessing signal peptides, which are not bound to the membrane. Cytochrome-containing nitrate reductase subunit NapM (FHQ18_08720), cytochrome *c* peroxidase (FHQ18_00480), and the cytochrome *c* oxidase *cbb3*-type subunit (FHQ18_11250) most probably are not involved in Fe(III) reduction. The functions of the other multiheme *c*-type cytochromes are not clear. Nine cytochrome genes, including those encoding proteins with 28 and 10 hemes, together with the other six genes of periplasmatic or membrane-bound proteins, are located in a short genome region (FHQ18_02900 - FHQ18_02990) and arranged in the same order as the genes of non-iron-reducing *D. desulfuricans* (DEFDS_0741-60), while the respective proteins have 49%–82% sequence identity with their *D. desulfuricans* counterparts. This gene cluster in *D. desulfuricans* was proposed as a remnant of the metal reduction system [9]. It should be noted that the gene cluster with a high content of cytochromes in the *D. autotrophicus* genome is adjacent to the gene cluster encoding [Ni-Fe]-containing hydrogenase (FHQ18_02875- FHQ18_02890), but in *D. desulfuricans*, the cytochrome and hydrogenase clusters are separated. This probably affects the co-expression of proteins involved in lithoautotrophic iron reduction. Only two genes of multiheme *c*-type cytochromes of *D. autotrophicus* have no homologs in *D. desulfuricans* genome: FHQ18_08740, encoding decaheme transmembrane cytochrome, and FHQ18_05250, encoding periplasmic octaheme cytochrome (Otr). Although we propose that these enzymes are involved in nitrite-reduction, it could not be excluded that they also participate in Fe(III) reduction. Similar to the genome of *D. desulfuricans,* the *D. autotrophicus* genome contains both the type IV pilin PilA (‘e-pilin’) (FHQ18_03575) and ‘pilA-C’ (FHQ18_03580) genes together in a cluster. FHQ18_03575 encodes a 65 aa immature pilin (57 aa mature pilin, 8 aa leader sequence) with eight aromatic amino acids (Supplementary Figure S1). This is the shortest ‘e-pilin’ reported to date. Other genes required for pilin assembly are scattered throughout the genome in five other clusters (FHQ18_00025–FHQ18_00040, FHQ18_00230–FHQ18_00275, FHQ18_04500–FHQ18_04520, FHQ18_06410–FHQ18_06420, FHQ18_09895–FHQ18_09915). The *D. autotrophicus* genome did not contain genes encoding the proteins of flavin-based extracellular electron transfer in Gram-positive bacteria [69].

Thus, the biochemical system of Fe(III) reduction in *D. autotrophicus* remains unresolved. Genomes of both microorganisms, iron-reducing *D. autotrophicus* and non-iron-reducing *D. desulfuricans*, have homologous gene clusters encoding the putative components of their Fe(III) reduction machinery—multiheme cytochromes and electrically conductive pili. Among representatives of the phylum *Deferribacteres* with sequenced genomes, the capacity for Fe(III) reduction was experimentally shown only for *D. autotrophicus*. Thus, at present, genomic data cannot be convincingly used to study iron reduction in other members of *Deferribacteres*.

### 3.7. Sulfur Reduction

*Deferribacter autotrophicus* couples organotrophic growth with a reduction of elemental sulfur but does not utilize sulfate or thiosulfate as the electron acceptors [5]. The enzymatic elemental sulfur reduction in prokaryotes proceeds either directly or indirectly via a soluble polysulfide intermediate [70]. 

In *D. autotrophicus*, the sulfur respiration most likely occurs via the action of polysulfide reductases (Psr), as was already suggested for *D. desulfuricans* [9]. Psr preferably reduces soluble polysulfide, which forms spontaneously in aqueous solutions containing elemental sulfur and sulfide [71]. In deep-sea hydrothermal vents, polysulfides are particularly abundant and support the energy-generating respiratory processes of numerous thermophiles. 

**Figure 7:**
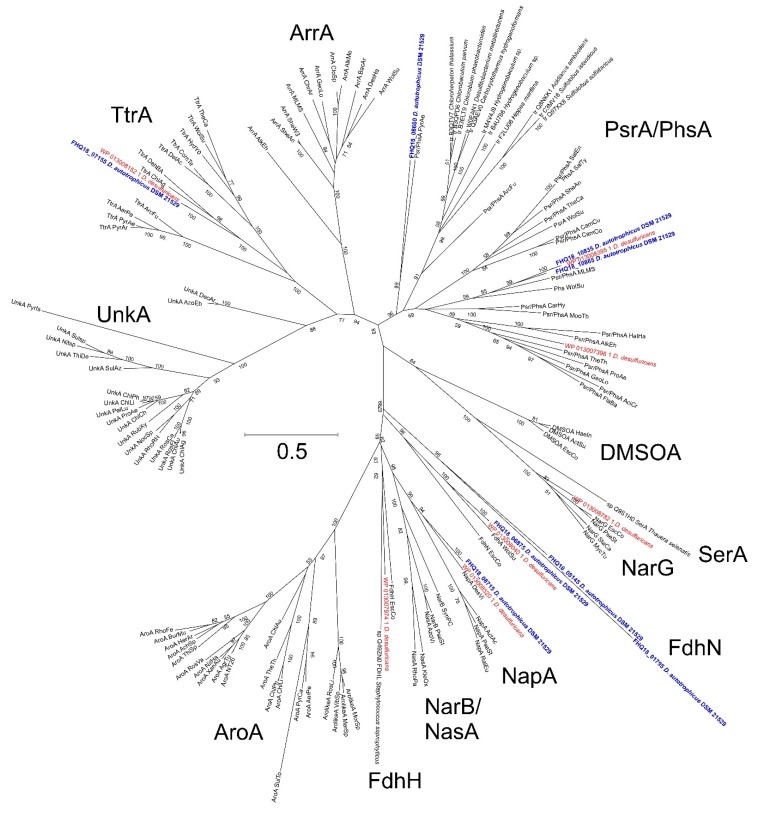
A maximum likelihood phylogenetic tree showing the position of *D. autotrophicus* (bold blue) and *D. desulfuricans* (red) molybdopterin oxidoreductase catalytic subunits A among the reference sequences [23], representing various molybdopterin protein families. The tree with the highest log likelihood is shown. The percentages of trees in which the associated taxa clustered together (bootstrap values, 1000 replicates) are shown next to the branches. This tree was inferred in MEGA6. The catalytic subunit clusters shown are: PsrA/PhsA, polysulfide reductase/thiosulfate reductases; AroA, arsenite oxidases; Arr, arsenate reductases; DMSOA, dimethylsulfoxide reductases; FdhN and FdnH, formate dehydrogenases; NapA, periplasmic nitrate reductases; NarG, membrane-bound nitrate reductases; NarB/NasA, assimilatory nitrate reductases (Nas); TtrA, tetrathionate reductases; SerA, selenate reductase; and UnkA, unknown reductases. Branch lengths (see the scale) correspond to the number of substitutions per site with corrections associated with the evolutionary model.

The *D. autotrophicus* genome has three gene clusters encoding polysulfide reductases (FHQ18_08590-FHQ18_08600; FHQ18_10855-FHQ18_10865; FHQ18_10825-FHQ18_10835). The catalytic subunits (PsrA) in all clusters are members of the molybdopterin superfamily (Psr/Phs) [72,73]. Genes for PsrABC subunits were located in the order typical for other *psr/phs*. No genes encoding the TorD maturation protein and rhodanese-like sulfur-transferases were found in close vicinity to the *psr* clusters. In addition to polysulfide reductases, the genome also encompasses a molybdopterin-containing tetrathionate reductase (Ttr), a protein, which reduces tetrathionate to thiosulfate (FHQ18_07155), but the functionality of this enzyme is uncertain due to the absence of an anchoring subunit TtrC. The phylogenetic analysis of subunits A of all eight *D. autotrophicus* molybdopterin proteins (Figure 7) suggests that the FHQ18_08590 - FHQ18_08600 Psr forms a deep lineage within the Psr branch, thereby indicating its ancient origin or rather old horizontal transfer from *Archaea*. The other two *psr* clusters (FHQ18_10855-FHQ18_10865; FHQ18_10825-FHQ18_10835) were recently (after the splitting of *D. autotrophicus* and *D. desulfuricans*) duplicated, which is also supported by their close location within a genomic context. *D. desulfuricans* has two Psr complexes. One is closely related to the duplicated FHQ18_10855-FHQ18_10865; FHQ18_10825-FHQ18_10835, while the other is located in the branch of well-characterized Psr enzymes and has no close homologs in *D. autotrophicus* (Figure 7). Homologs of bacterial porin (FHQ18_02515) and rhodanese-like sulfur-transferase (FHQ18_07165), encoded by the genes located close to the *Ttr* cluster, could be involved in the entrance of polysulfide into periplasmic space. The genome of *D. autotrophicus* did not contain the homologs of NAD(P)H elemental sulfur reductase (NSR) [74], H_2_:sulfur oxidoreductase, or sulfhydrogenase [75] that were previously proposed to be involved in sulfur reduction in hyperthermophilic archaea and bacteria. It should be noted that later studies have shown that NSR is not essential for energy conservation during S^0^ reduction [76], while sulfhydrogenase is down-regulated in the presence of S^0^ [77].

Although, the genomes of all representatives of the phylum *Deferribacteres*, except *Mucispirillum schaedleri*, contain from one to three gene clusters related to polysulfide reductase PsrABC (Table 2), none of them besides *D. autotrophicus*, *D. desulfuricans*, and *Geovibrio thiophilus* are capable for S^0^ reduction [44]. Probably, these molybdopterin enzymes participate in the reduction of different electron acceptors in other *Deferribacteres. D. autotrophicus* is incapable for sulfide oxidation [5]; most likely, the additional PSRs cannot act in the reversible direction as sulfide dehydrogenase, which was suggested for some *Epsilonproteobacteria* with several *psr* copies [78].

## 4. Conclusions

Genomic analysis of *D. autotrophicus* SL50^T^ revealed several new remarkable metabolic traits of this bacterium and the *Deferribacteres* phylum. Genomic data suggest that CO_2_ assimilation is carried out via a recently discovered reversible TCA cycle. We predicted and experimentally proved the capability of *D. autotrophicus* to grow by means of the oxidation of carbon monoxide. CO oxidation occurred only with nitrate as an electron acceptor, which was reduced to ammonium. Utilization of carbon monoxide most likely involves anaerobic [Ni, Fe]-containing carbon-monoxide dehydrogenase, encoded in the *rcoM-cooS-cooC-cooF-FNOR* gene cluster. This is the first evidence of CO metabolism in the phylum *Deferribacteres*. The nitrate reduction system is represented by the NapMADGH complex. The conversion of nitrite to ammonium proceeds via a non-canonical putative pathway based on the action of hydroxylamine oxidoreductase (Hao) and hydroxylamine reductase (Hcp). The biochemical system of Fe(III) reduction in *D. autotrophicus* remains unresolved. The *D. autotrophicus* genome contains 17 genes encoding putative multiheme *c*-type cytochromes and ‘e-pilin’ genes, some of which are probably involved in Fe(III) reduction. Elemental sulfur reduction in *D. autotrophicus* presumably occurs due to the action of Psr/Phs-type molybdopterin enzymes, encoded in three *psrABC* gene clusters. 

## Figures and Tables

**Table 1:** Genomic features of *D. autotrophicus* SL50^T^ and *D. desulfuricans* SSM1.

	*D. autotrophicus* SL50^T^	*D. desulfuricans* SSM1 [9]	
	Chromosome Contigs *	Chromosome	Plasmid
Size	2,543,746	2,234,389	308,544
GC-content	32.6	31.1	24.5
Protein coding genes			
total	2451	2396	287
pseudogenes	23	12	0
RNA genes	53		
rRNA operons	2	2	-
tRNA	42	44	-
small ncRNA	4	2	-
IS elements	66 *	7	34
IS element families	IS110, IS21, IS256, ISL3, IS3, IS5, IS1634, IS607	IS256, IS5, ISL3	IS200/IS605, IS256, IS5
CRISPR-Cas systems			
CRISPR repeat stretches	3	1	1
CRISPR spacers	26		
Complete Cas gene clusters	0	1	1
Cas-related genes	5		
Genomic islands			
Number of GIs	12	5	-
Total length of GIs	164,610	72,606	-

* Data in Table 1 refer to draft genome assembly of *D. autotrophicus*. Therefore, there is a possibility that data in the table does not represent the full set of tRNA genes and IS elements.

**Table 2:** Dissimilatory nitrate and elemental sulfur reduction and related genes in genome-sequenced *Deferribacteres*.

Microorganism ^1^	Nitrate Reduction	Product	*napMADGH*	*narGHJI*	*nrfA*	*hao*	*hcp*	*otr*	S^0^ Reduction	*psrABC*
*Calditerrivibrio nitroreducens*	+	NH_4_^+^	+	-	+	-	+	-	-	+
*Deferribacter autotrophicus*	+	NH_4_^+^	+	-	-	+	+	+	+	+++
*Deferribacter desulfuricans*	+	NO_2_^−^	+	+	-	+	+	-	+	+++
*Denitrovibrio acetiphilus*	+	NH_4_^+^	+	+	-	+	+	-	-	++
*Flexistipes sinusarabici*	nt	nt	+	-	-	+	+	-	-	++
*Geovibrio thiophilus*	+	NO_2_^−^, NH_4_^+^	+	+	-	-	+	-	+	++
*Mucispirillum schaedleri*	-	-	+	-	+	-	-	-	nt	-
*Seleniivibrio woodruffii*	-	-	+	+	+	+	+	-	-	+++

^1^ Data are presented for the type strains; nt—not tested. Gene abbreviations: *nap*—periplasmic nitrate reductase, *nar*—nitrate reductase, *nrfA*—ammonifying nitrite reductase, *hao*—hydroxylamine oxidoreductase, *hcp*—hydroxylamine reductase, *otr*—octaheme tetrathionate reductase, and *psrABC*—polysulfide reductase (the number of + corresponds to the number of *psrABC* clusters).

## Supplementary Materials

The following are available online at https://www.mdpi.com/2073-4425/10/11/849/s1, Figure S1: Protein sequences of type IV pilin PilA (‘e-pilin’) from *Deferribacter autotrophicus* SL50^T^, *Deferribacter desulfuricans* SSM1^T^ and *Geobacter metallireducens* GS-15^T^. Table S1. Orthologs of *D. autotrophicus* chromosome and *D. desulfuricans* chromosome and plasmid. Table S2: Genomic islands of *Deferribacter autotrophicus*. Table S3: COGs of the genomic islands of *Deferribacter autotrophicus*. Table S4. Genes related to signal transduction systems in genomes of *D. autotrophicus* and *D. desulfuricans*. Table S5: Best Blast Hit species in the genomic islands of *Deferribacter autotrophicus*. Table S6: Multiheme c-type cytochromes encoded in the genome of *Deferribacter autotrophicus*.
