# Supplementary material for: Genomic Insights into the Carbon and Energy Metabolism of a Thermophilic Deep-Sea Bacterium Deferribacter autotrophicus Revealed New Metabolic Traits in the Phylum Deferribacteres
Source: Genes (Basel). 2019 Oct 26;10(11):849. doi: 10.3390/genes10110849 (PMC6896113; doi:10.3390/genes10110849)
Supplement: Supplementary file 1 [file genes-10-00849-s001.zip › Figure S1.pptx]

## Slide 1
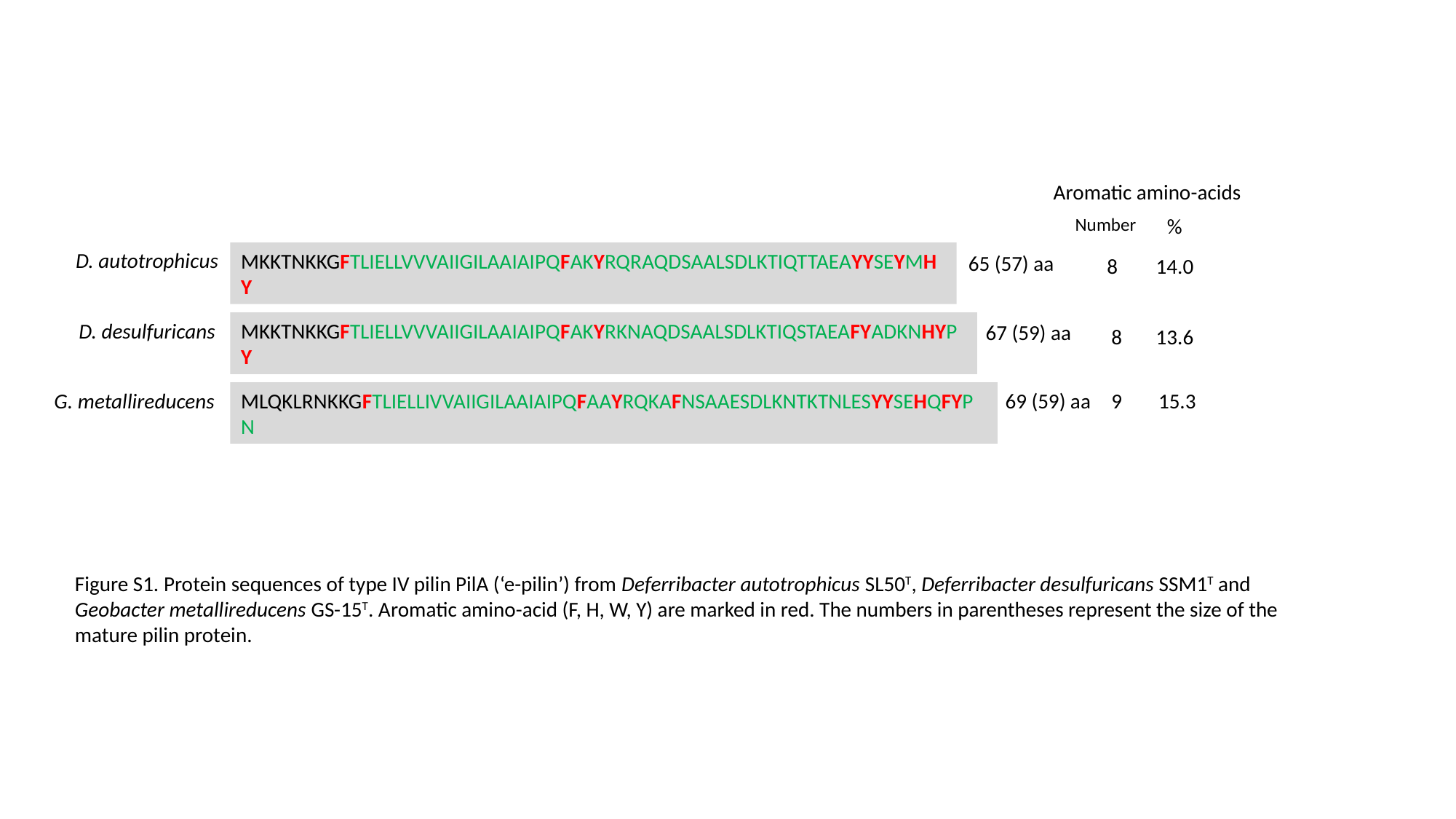

Aromatic amino-acids
%
Number
D. autotrophicus
MKKTNKKGFTLIELLVVVAIIGILAAIAIPQFAKYRQRAQDSAALSDLKTIQTTAEAYYSEYMHY
65 (57) aa
14.0
8
D. desulfuricans
mkktnkkgftliellvvvaiigilaaiaipqfakyrknaqdsaalsdlktiqstaeafyadknhypy
67 (59) aa
8
13.6
69 (59) aa
G. metallireducens
mlqklrnkkgftliellivvaiigilaaiaipqfaayrqkafnsaaesdlkntktnlesyysehqfypn
9
15.3
Figure S1. Protein sequences of type IV pilin PilA (‘e-pilin’) from Deferribacter autotrophicus SL50T, Deferribacter desulfuricans SSM1T and Geobacter metallireducens GS-15T. Aromatic amino-acid (F, H, W, Y) are marked in red. The numbers in parentheses represent the size of the mature pilin protein.
