# Supplementary material for: Genomic Insights into the Carbon and Energy Metabolism of a Thermophilic Deep-Sea Bacterium Deferribacter autotrophicus Revealed New Metabolic Traits in the Phylum Deferribacteres
Source: Genes (Basel). 2019 Oct 26;10(11):849. doi: 10.3390/genes10110849 (PMC6896113; doi:10.3390/genes10110849)
Supplement: Supplementary file 1 [file genes-10-00849-s001.zip › Supplementary Table S6.docx]

Table S4. Multiheme c-type cytochromes encoded in genome of *Deferribacter autotrophicus*.

| Locus tag | Number of heme c-binding motifs | Length, aa | Predicted cellular location | Gene annotation (IMG) | % aa identity with homolog in *D. desulfuricans* |
| --- | --- | --- | --- | --- | --- |
| FHQ18_02925 | 28 | 987 | periplasm | predicted CXXCH cytochrome family protein | 58 |
| FHQ18_04550 | 11 | 507 | TM* | nitrate/TMAO reductase-like tetraheme cytochrome c subunit | 85 |
| FHQ18_02990 | 10 | 512 | TM | predicted CXXCH cytochrome family protein | 82 |
| FHQ18_08740 | 10 | 640 | TM | hypothetical protein | 0 |
| FHQ18_02940 | 8 | 317 | cytoplasm | predicted CXXCH cytochrome family protein | 68 |
| FHQ18_08725 | 8 | 531 | periplasm | multi-heme cytochrome with CxxCH motif | 89 |
| FHQ18_05250 | 8 | 492 | periplasm | cytochrome c (Cluster: Cytochrome_cB) | 0 |
| FHQ18_02905 | 6 | 341 | TM | hypothetical protein | 78 |
| FHQ18_02900 | 5 | 330 | periplasm | predicted CXXCH cytochrome family protein | 49 |
| FHQ18_02960 | 4 | 201 | TM | predicted CXXCH cytochrome family protein | 78 |
| FHQ18_02965 | 4 | 228 | TM | doubled CXXCH motif protein | 76 |
| FHQ18_02970 | 4 | 154 | TM | predicted CXXCH cytochrome family protein | 54 |
| FHQ18_08720 | 3 | 117 | periplasm | periplasmic nitrate reductase subunit NapM | 74 |
| FHQ18_00480 | 2 | 318 | TM | cytochrome c peroxidase | 0 |
| FHQ18_12220 | 2 | 350 | cytoplasm | hypothetical protein | 0 |
| FHQ18_02915 | 2 | 274 | TM | hypothetical protein | 74 |
| FHQ18_11250 | 2 | 295 | TM | cytochrome c oxidase cbb3-type subunit 2 | 0 |

*TM – transmembrane
